# Severe Rhabdomyolysis Complicated With Acute Kidney Injury Required Renal Replacement Therapy After Pfizer COVID-19 Vaccine  

**DOI:** 10.7759/cureus.25199

**Published:** 2022-05-22

**Authors:** Turki A Banamah, Anas A Bogari, Alfaisal Neyazi, Eman Kotbi, Hatim Almaghraby, Firas Atwah

**Affiliations:** 1 Nephrology and Kidney Transplant, King Abdulaziz Medical City Jeddah, Jeddah, SAU; 2 Nephrology and Kidney Transplantation Consultant, National Guard Health Affairs, Jeddah, SAU; 3 Internal Medicine, King Abdulaziz Medical City Jeddah, Jeddah, SAU; 4 Internal Medicine, King Abdullaziz Medical City Jeddah, Jeddah, SAU; 5 Nephrology, King Abdulaziz Medical City Jeddah, Jeddah, SAU; 6 Pathology, King Abdullah International Medical Research Centre (KAIMRC), Jeddah, SAU; 7 Pathology, King Abdulaziz Medical City Jeddah, Jeddah, SAU

**Keywords:** pfizer-biontech covid-19 vaccine, covid-19 vaccine complication, rhabdomyolysis with acute renal failure, vaccine induced rhabdomyolysis, covid 19

## Abstract

The adverse effects of coronavirus disease 2019 (COVID-19) vaccines are somewhat common but rarely life-threatening. Diagnosing life-threatening vaccine-related adverse effects is heavily dependent on history taking and ruling out the other possible causes. Vaccine-related complications vary, so awareness of possible complications can lead to efficient management. We present the case of a 58-year-old woman with a history of schizophrenia who received the COVID-19 Pfizer vaccine and developed severe rhabdomyolysis. She required renal replacement therapy and fully recovered with possible transient autoimmune activity. This case highlights the importance of early awareness of adverse effects following vaccine administration and careful history taking and monitoring to avoid life-threatening conditions.

## Introduction

Vaccines are vital for preventing disease spread during the coronavirus disease 2019 (COVID-19) pandemic [1]. The World health organization approved several COVID-19 vaccines for "Emergency Use Instructions" [1]. 

Since their development, adverse effects were reported in the literature, such as mild symptoms like local site injection pain, fever, headache, and life-threatening adverse effects, including but not limited to myocarditis, vaccine-induced thrombocytopenia, and rhabdomyolysis [2].  

Rhabdomyolysis is a pathological process of muscle cell injury that causes the release of cell products into the circulation, manifesting as muscle weakness, myalgia, and swelling. Markers of muscle damage like creatine kinase and myoglobinuria are frequently present. Severe cases might result in acute kidney injury (AKI), which may require dialysis [i.e., renal replacement therapy (RRT)] in rare cases [3]. 

Although rhabdomyolysis was reported as an adverse effect of the COVID-19 vaccine, it usually presents with mild symptoms [4-6]. A previous case report also demonstrated rhabdomyolysis that didn’t require RRT after a patient received the Moderna vaccine [7]. We report a case of rhabdomyolysis in a patient who received the Pfizer COVID-19 vaccine and developed AKI requiring RRT. 

## Case presentation

A 58-year-old woman with a history of schizophrenia for more than ten years with no prior hospitalization or significant surgeries presented to the emergency department (ED) and reported generalized weakness and lethargy that started one day before her presentation. She reported that while taking a shower, she started to experience lower limb weakness on both proximal and distal muscles simultaneously and had to sit down. Her symptoms then progressed rapidly, and she could not stand or sit by the end of the day. The patient is unemployed, single, and lives in the same building with her brother. She has no family history of autoimmune diseases or other significant disorders. She is treated at the psychiatry clinic with regular follow-up for schizophrenia, controlled on flupenthixol 40 mg intramuscular every two weeks, trifluoperazine sustained release 5 mg orally once daily, and benztropine mesylate 2 mg orally once daily.  

She received a third Pfizer COVID-19 vaccine dose (i.e., the booster dose) the morning of the day her symptoms began. She experienced no neurological symptoms, no arthralgia, or myalgia. She developed a decrease in urine output with tea-colored urine and mild dysuria but no flank pain, fever, or chills. She denied any history of recent illness exposure, and all other systemic review findings were unremarkable. There was no record of new medication use, and she denied a history of alcohol intake or illicit drug use. She had no history of trauma or recent strenuous exercise.  

 When she presented to ED, she was conscious, oriented, and lethargic. Her vital signs were within reference ranges. Her neurological examination revealed power 3/5 in all proximal and distal muscles, normal tone, and reflexes with intact sensation. Additional exam findings were unremarkable except for a bruise on her right arm at the vaccine injection site, but she had no signs of swelling, arthritis, or cellulitis.  

Her laboratory evaluation revealed elevated creatinine, creatine kinase, and blood urea nitrogen levels. She had elevated anion-gap metabolic acidosis and severe hyperkalemia and was positive for urine myoglobin. She had mild hypocalcemia, hyperphosphatemia, hyperuricemia, and lactic acidosis with high inflammatory markers. Her hemoglobin and hematocrit levels were within reference ranges. She had leukocytosis (mainly neutrophils). Her liver function tests showed a hepatocellular injury pattern with significant aspartate transaminase and alanine transaminase elevation, a mild rise in alkaline phosphatase, and gamma-glutamyl transferase (GGT), while bilirubin and coagulation tests results (including international normalized ratio and prothrombin time) were within reference ranges. Urine analysis revealed low red blood cell count, high white blood cell count, micro proteinuria, positive leukocyte esterase, negative nitrate, and positive calcium oxalate crystals. Her urine toxicology screen was negative, and drug levels for acetaminophen and salicylate were within reference ranges. Her thyroid function test result was unremarkable. Table 1 presents selected laboratory results on admission through discharge.  

**Table 1:** Laboratory Evaluations during patient hospitalization Abbreviations: RRT, renal replacement therapy; BUN, blood urea nitrogen; CK, creatine kinase.

Test	On Admission	RRT Initiation (Week 1)	After Halting RRT (Week 3)	Recovery Phase (Week 5)	On Discharge (Week 6)	Reference Ranges
Sodium (mmol/L)	138	129	136	140	141	135-145
Potassium (mmol/L)	6.3	3.7	4.1	4.1	4.2	3.5-4.9
Bicarbonate (mmol/L)	15	15	25	25	26	22-29
Creatinine (µmol/L)	239	758	470	136	117	50-74
BUN (mmol/L)	12.2	29.3	12.1	11.7	8	1.9-5.7
CK (IU/L)	42,670	9202	93	36	29	27-132

The patient was admitted as a case of severe AKI secondary to rhabdomyolysis and was started on intravenous (IV) fluid with strict monitoring of intake and output. We suspected neuroleptic malignant syndrome based on her history and use of antipsychotic medications. However, she did not meet the criteria for diagnosis. We sent for a laboratory workup for causes of rhabdomyolysis and inflammatory myopathies. Her autoantibodies were negative except for the Mi-2 beta antibody.  

**Figure 1:**
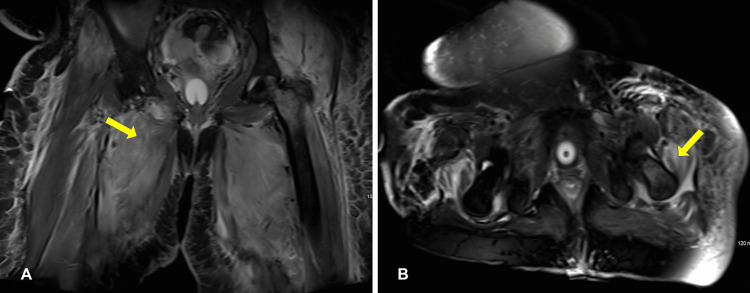
Magnetic Resonance Image of her Thigh and Femur Generally showed diffuse subcutaneous edema bilaterally, Some left femoral bone marrow edema in the neck and trochanteric region could represent transient osteoporosis versus bone contusion. No cortical bone destruction. (A) Coronal view of pelvic T1 weighted MRI with increased signal intensity of adductor longus muscle (arrow). (B) Axial view of pelvic T1 weighted MRI with increased signal intensity of gluteus medius tendon (arrow). All findings were suggestive of myositis. Abbreviation: MRI, magnetic resonance imaging.

The magnetic resonance image of her thigh and femur (Figure 1) showed features suggestive of polymyositis, with increased signal intensity of the adductors bilaterally and gluteal medius and intermedius bilaterally with diffuse subcutaneous edema. Her electromyography revealed generalized myopathic motor units in the proximal and distal muscles in the lower and upper extremities. Motor nerve conduction studies revealed no abnormalities. The overall clinical features and course of the disease did not support a diagnosis of inflammatory myopathies.  

**Figure 2:**
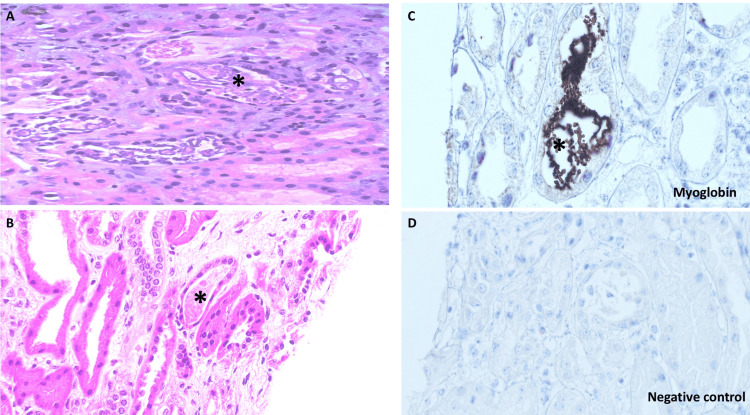
Kidney Biopsy Results with Negative Control Comparison (A&B) Renal proximal tubules with degenerative epithelial changes and irregular red-brown tubular casts with sharply defined edges (asterisk). The interstitium shows moderate mixed chronic inflammation and edema, x400 and x200, respectively (H&E stain). (C) Tubular casts demonstrate positive immunoreactivity for myoglobin (asterisk) by immunohistochemistry, x400, DAB, Ventana BenchMark Ultra (Roche Diagnostics, Basel, Switzerland). (D) Negative control immunohistochemistry slide demonstrating tubular casts with negative immunoreactivity, x400, DAB, Ventana BenchMark Ultra. Abbreviation: H&E, hematoxylin and eosin; DAB, diaminobenzidine.

Her renal function deteriorated despite supportive treatment, and she remained anuric, developed refractory metabolic abnormalities to medical therapy, and developed a picture of volume overload. We initiated RRT for her. In addition, the patient underwent renal biopsy (Figures 2A, 2B, 2C, 2D) that revealed diffuse tubular damage with degenerative and regenerative features with interstitial edema and fibrosis. We noted brown-colored angulated, sharply defined tubular casts, positive for myoglobin on immunohistochemistry evaluation. Her glomeruli immunofluorescence study was negative for antiserum directed against IgG, IgA, IgM, C3, C1q, kappa, and lambda. The tubular basement membrane and vessel wall were unremarkable.  

The patient received eight RRT sessions, with an assessment before each session for indications. Once she began to have good urine output, we withheld her RRT. She entered the polyureic recovery phase, and her fluids were replaced according to total fluid loss. During the patient's hospital stay, she was diagnosed with COVID-19 based on a swab for screening before RRT line insertion, but a chest x-ray showed no infiltration. After six weeks of hospitalization, she was discharged home safely, without the need to continue her regular hemodialysis sessions. 

## Discussion

Vaccines were typically recognized to carry some risk of adverse effects, and the COVID-19 vaccine was no different. The majority of adverse effects of messenger ribonucleic acid vaccines like the Pfizer and Moderna COVID-19 vaccines were limited to injection site pain and swelling, flu-like symptoms, and headaches [8]. 

These effects may become more severe with exposure to the second dose of the vaccine. However, long-term data are still important for identifying other symptoms and adverse effects, given the relatively new method of making the COVID-19 vaccine [8].    

Several studies suggested a transient increase in patients' autoimmune activity after receiving the COVID-19 vaccine [9-13]. Six cases of rhabdomyolysis were observed in patients who received the COVID-19 vaccine [4-7,14,15], only two of whom experienced AKI like our patient, and both were related to Moderna Vaccine [7,15]. 

In one case, the AKI resolved with hydration and did not require RRT [7]. The other AKI patient's condition worsened due to late presentation to the hospital with concurrent heart failure, which prevented full hydration treatment. The patient required RRT but developed cardiac arrest and anoxic brain injury and subsequently died [15].  

Our patient was on three antipsychotic medications for more than ten years to treat her schizophrenia. Antipsychotic medications can increase the risk of rhabdomyolysis and neuroleptic malignant syndrome, so monitoring those patients' creatine kinase levels is important [16]. 

However, none of our patient's medications were associated with rhabdomyolysis. Also, she did not respond much to IV fluid hydration despite the high infusion rate. It is unclear why her rhabdomyolysis did not improve with hydration or why she continued to have myositis symptoms one week from the COVID-19 vaccine dose. 

Her response to RRT and recent COVID-19 vaccine history showed that her rhabdomyolysis was likely COVID-19 vaccine-related. 

## Conclusions

This case described a 58-year-old woman who received the COVID-19 Pfizer vaccine and developed severe rhabdomyolysis that was resolved on RRT with possible transient autoimmune activity. This case highlights the importance of early awareness of adverse effects following vaccine administration and careful history taking. Thus, patients who present to the ED require careful monitoring to avoid life-threatening conditions. 
